# Supplementary figures and images for: The intrinsically disordered N-terminus of the voltage-dependent anion channel
Source: PLoS Comput Biol. 2021 Feb 12;17(2):e1008750. doi: 10.1371/journal.pcbi.1008750 (PMC7906469; doi:10.1371/journal.pcbi.1008750)

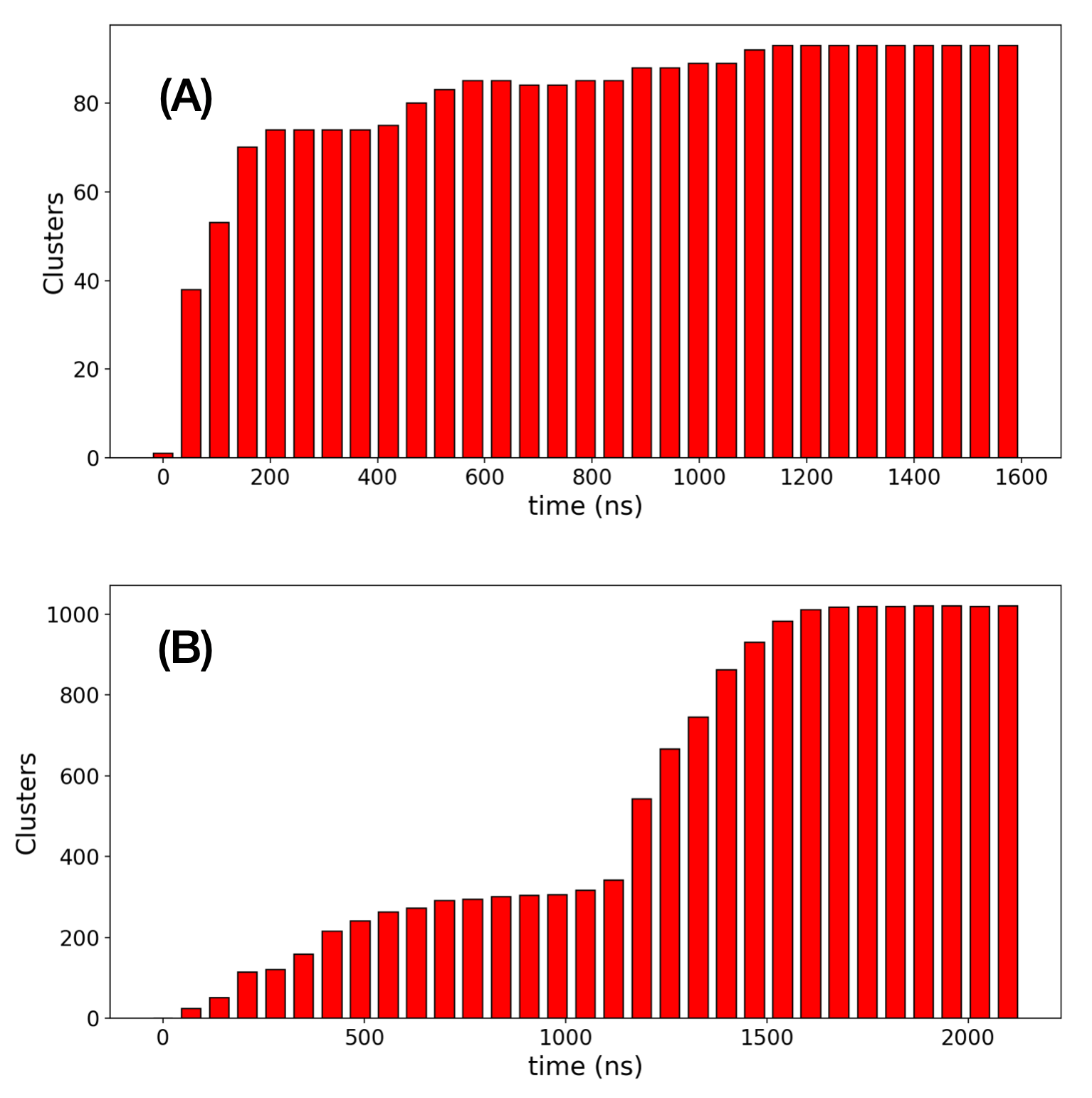

Supplement: S1 Fig — The figure shows the number of clusters generated as a function of the simulation time in the case of the ff14SB force field (A) and of the ff14IDPSFF force field (B). Clustering analysis was performed using a hierarchical agglomerative approach with an RMSD threshold of 3 Å. (TIF) [file pcbi.1008750.s001.tif]

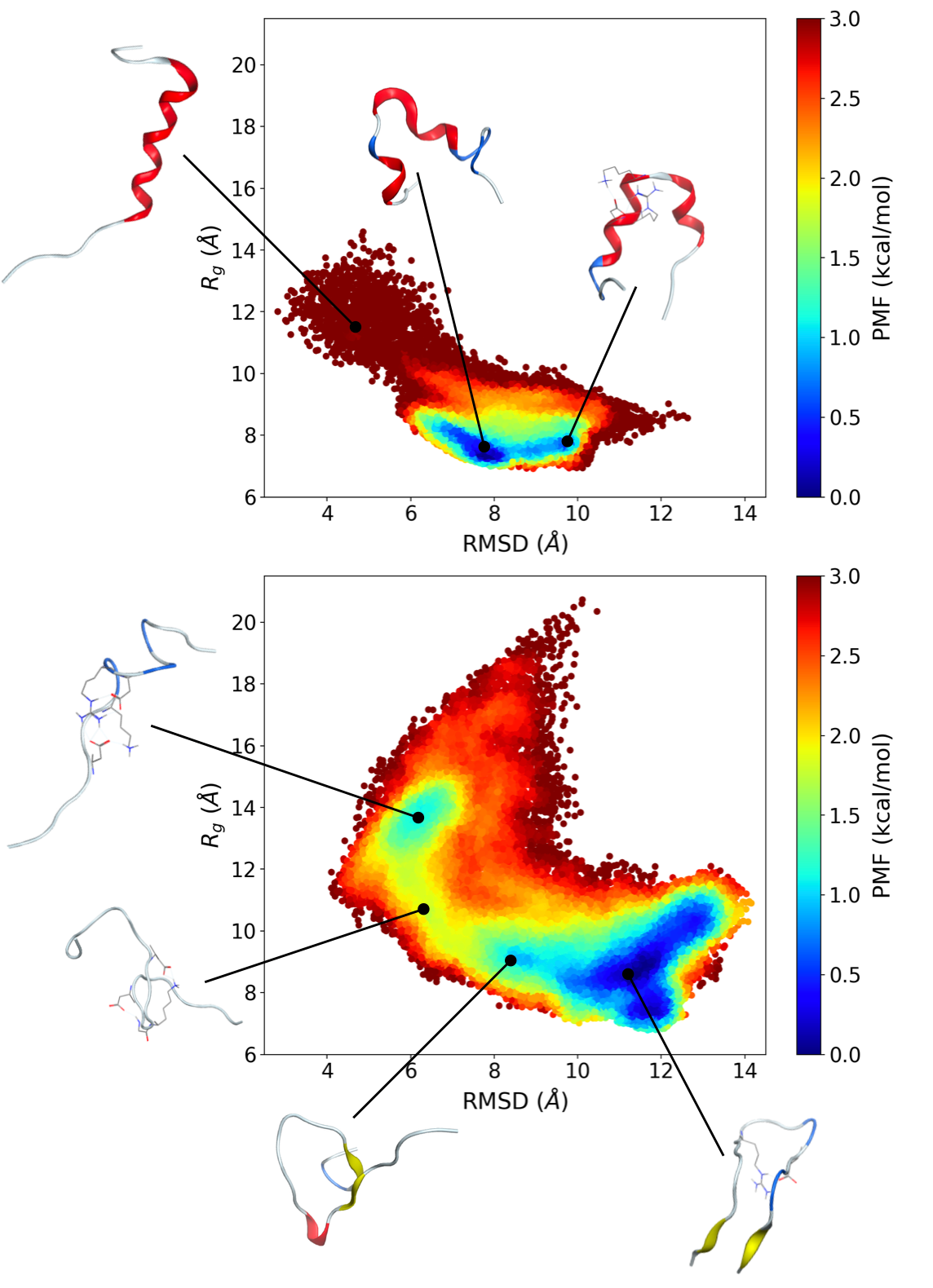

Supplement: S2 Fig — PMFs were obtained in the cases of ff14SB (top) and ff14IDPSFF (bottom) and are given as a function of the radius of gyration (Rg) and the RMSD with respect to the N-terminal peptide conformation in the 3EMN crystal structure. Representative structures in main basins and transition regions are also depicted. The top figure was built out of a 1.6 μs-long trajectory while data at the bottom were collected over 2.1 μs. (TIF) [file pcbi.1008750.s002.tif]

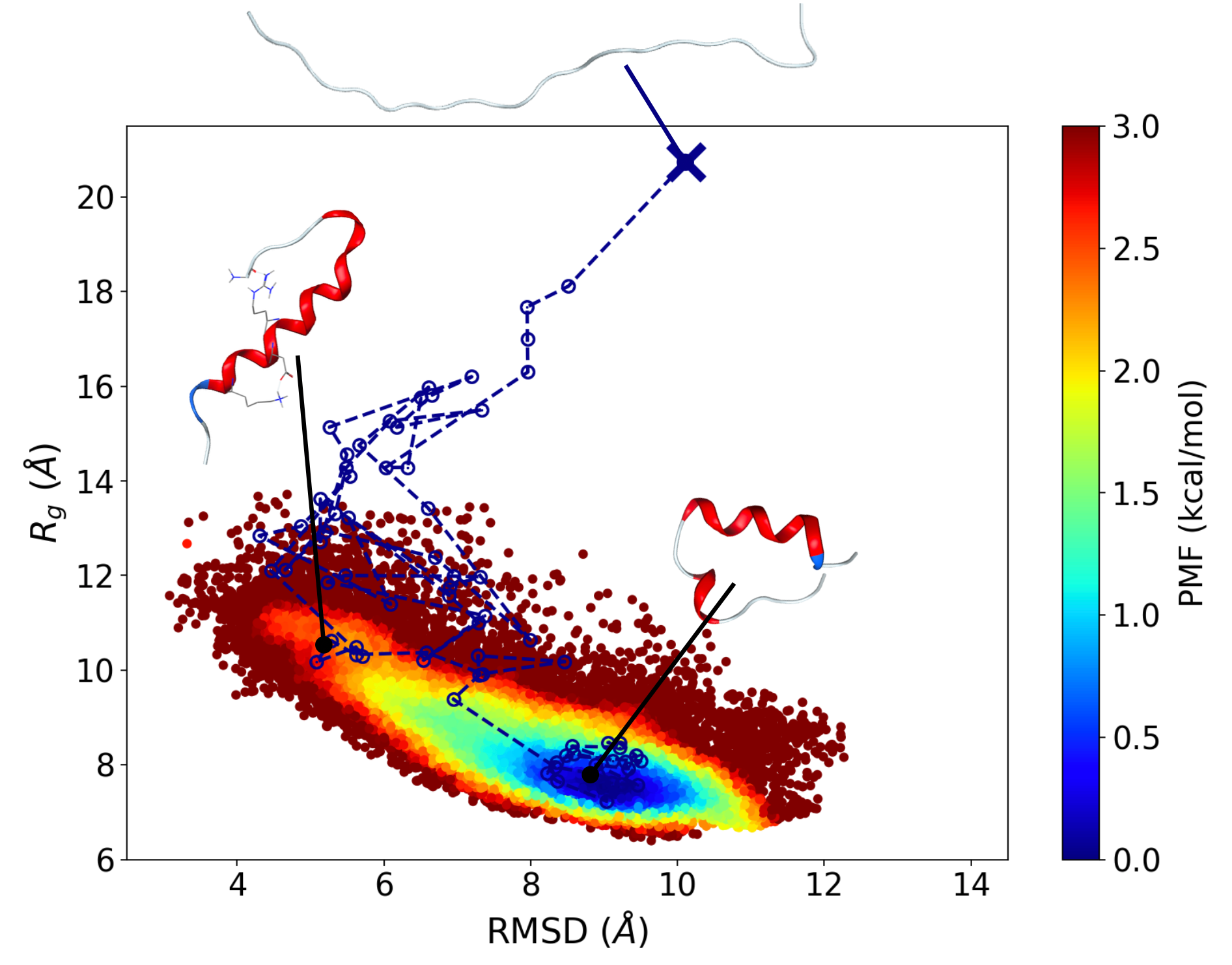

Supplement: S3 Fig — Due the biased nature of the simulation, density values were reweighted from a Maclaurin series up using terms up to rank k = 10 (Eq (5)). As in S2 Fig, the PMF is given as a function of the radius of gyration (Rg) and the RMSD with respect to the N-terminal peptide conformation in the crystal structure of mVDAC1. The dashed dark-blue trajectory represents a 20-ns-long aMD trajectory generated by initiating our simulation from a fully unfolded structure (dark blue cross) that clearly shows the convergence to the helix-enriched basin. (TIF) [file pcbi.1008750.s003.tif]

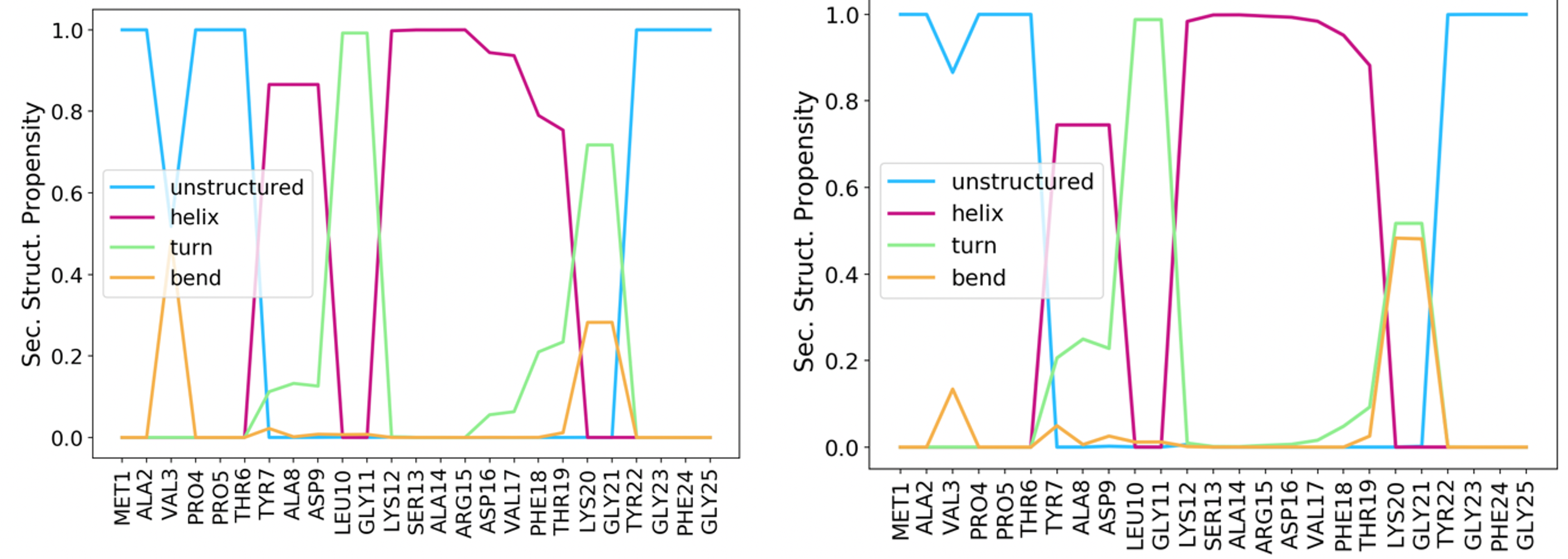

Supplement: S4 Fig — Values were obtained from 100-ns-long MD runs using the ff14SB force field (left) and the ff14IDPSFF force field (right). Note that β-content is not displayed as it is everywhere zero and helical content includes both 310-helical and regular α-helical content. For both force fields, 310-helical content is found only in the short helix made of residues Y7 to D9 and regular helix content was only observed in the long helix made of residues K12 to T19. (TIF) [file pcbi.1008750.s004.tif]

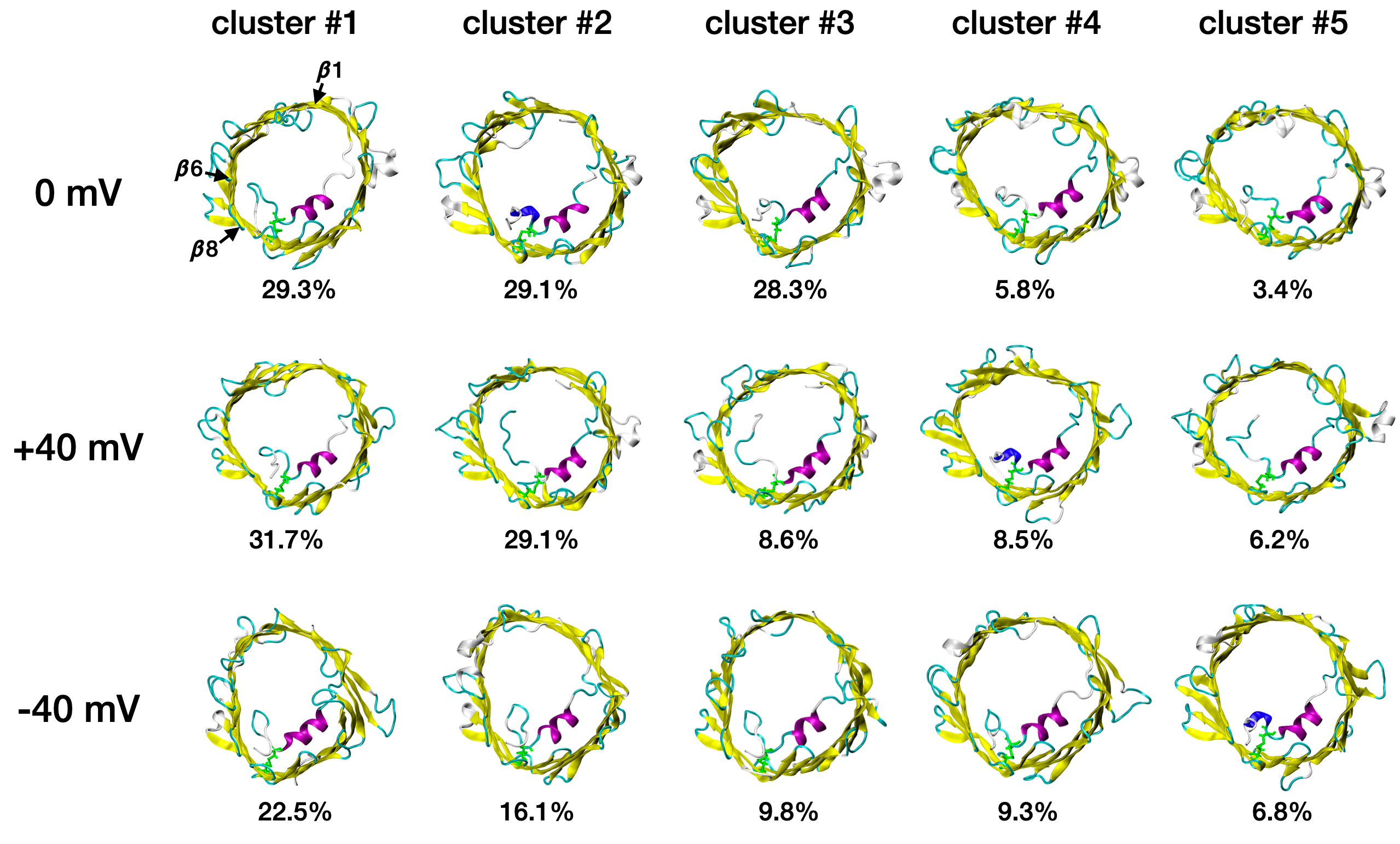

Supplement: S5 Fig — Conformers were obtained from agglomerative hierarchical clustering analysis of aMD trajectories using the RMSD of the N-terminus as a metric and a threshold distance of 3.0 Å. The representative structure (viewed from the IMS) of the 5 most populated clusters is shown at each voltage. Disulfide bridge linked L10C and A170C residues are shown in green. (TIF) [file pcbi.1008750.s005.tif]

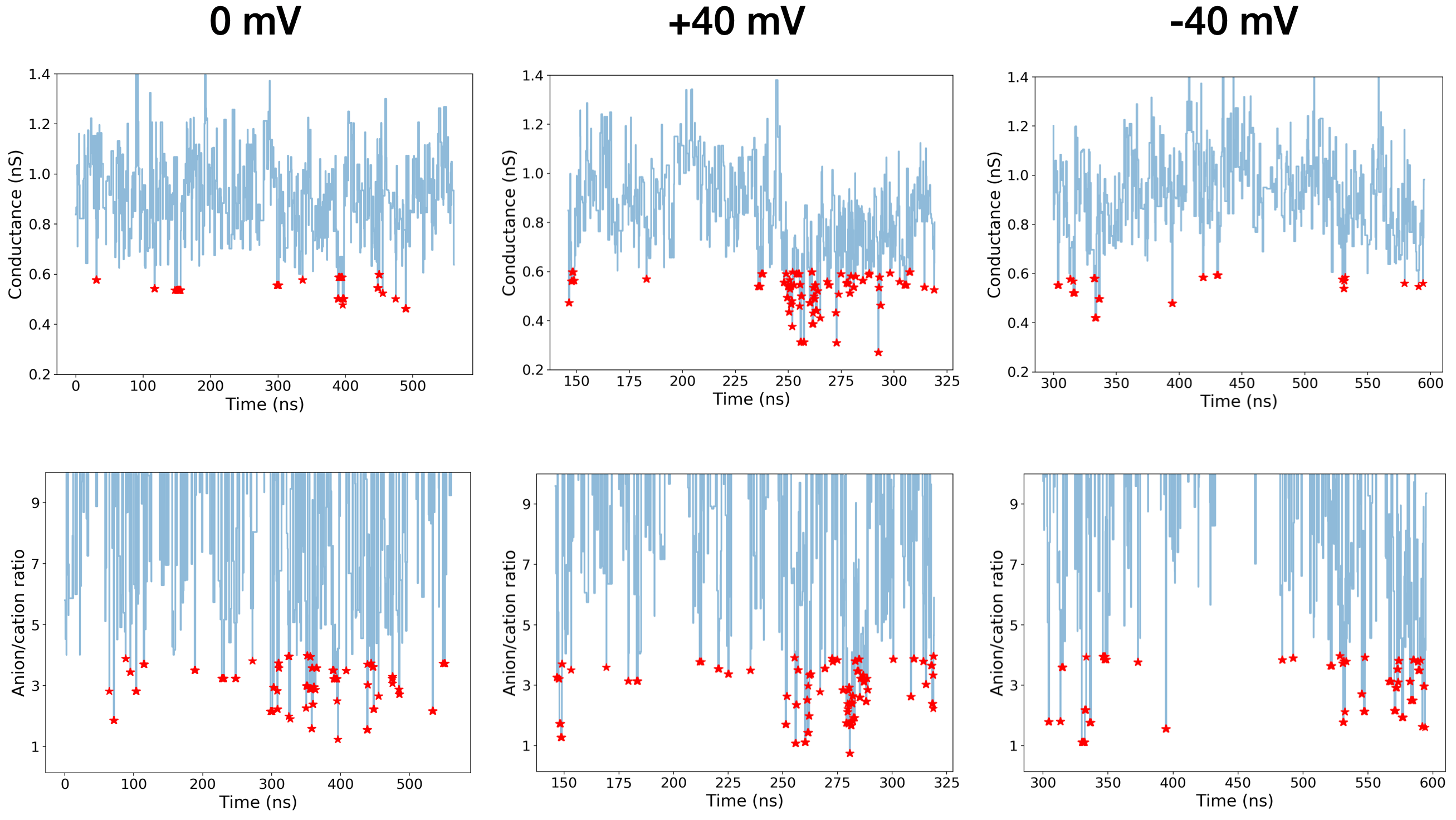

Supplement: S6 Fig — All values were obtained from GCMC/BD runs at 150-mM KCl concentration performed on 500 representative structures of each aMD trajectory. Time series curves were reconstructed by assigning the same conductance and anion/cation ratio to all the frames of a given cluster. Red stars correspond to all the frames with a conductance less than 0.6 nS or an anion/cation ratio less than 4. Note that the frames extracted from our aMD trajectories at +40mV and -40mV were selected from 140 ns and 300 ns, respectively. (TIF) [file pcbi.1008750.s006.tif]

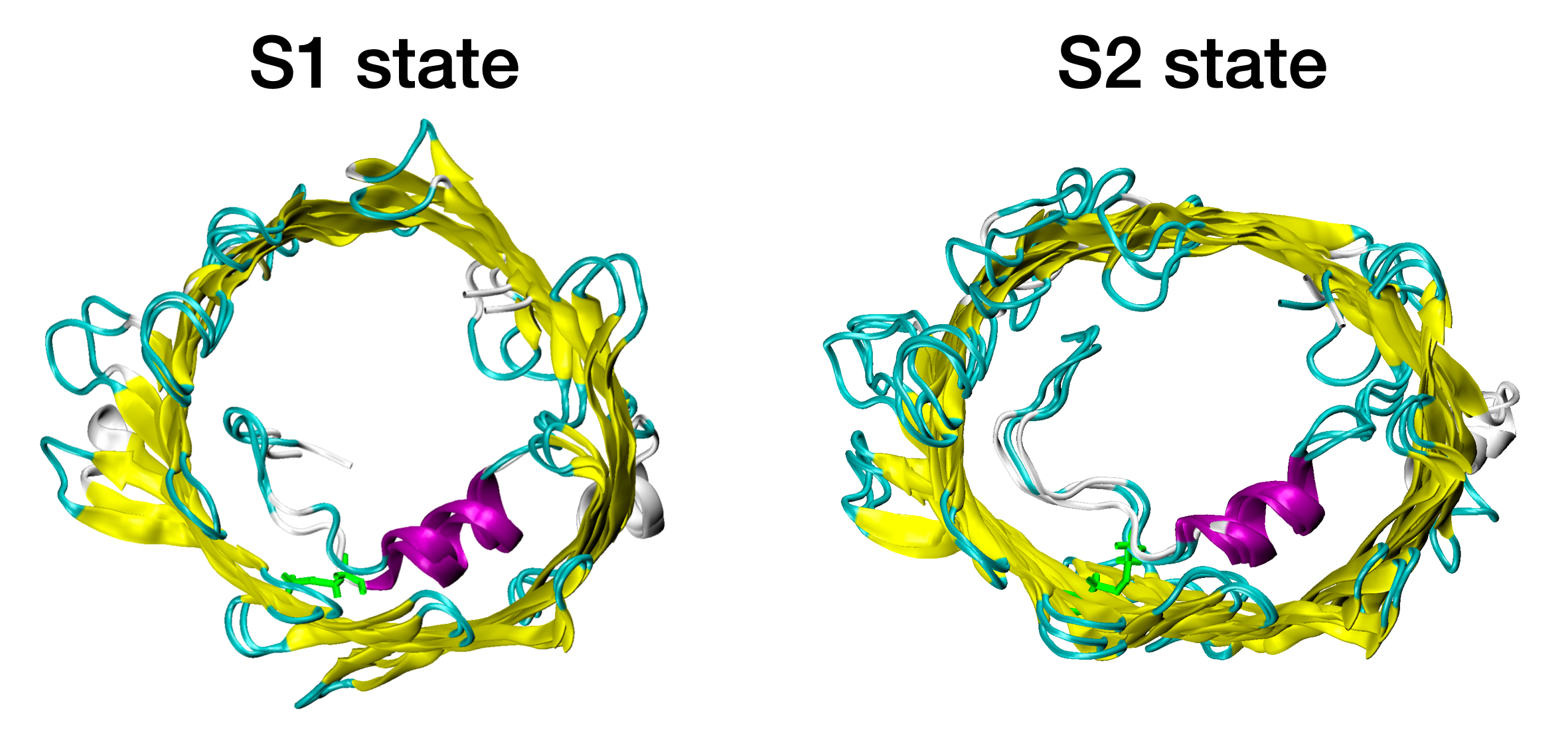

Supplement: S7 Fig — The frames (IMS view) correspond to the rows highlighted in green in S3 Table, namely frames 4, 12, 30, 37 and 46. The first cluster (S1 state) is made of frames 30 and 46 while the second cluster is made of frames 4, 12 and 37 (S2 state). (TIF) [file pcbi.1008750.s007.tif]

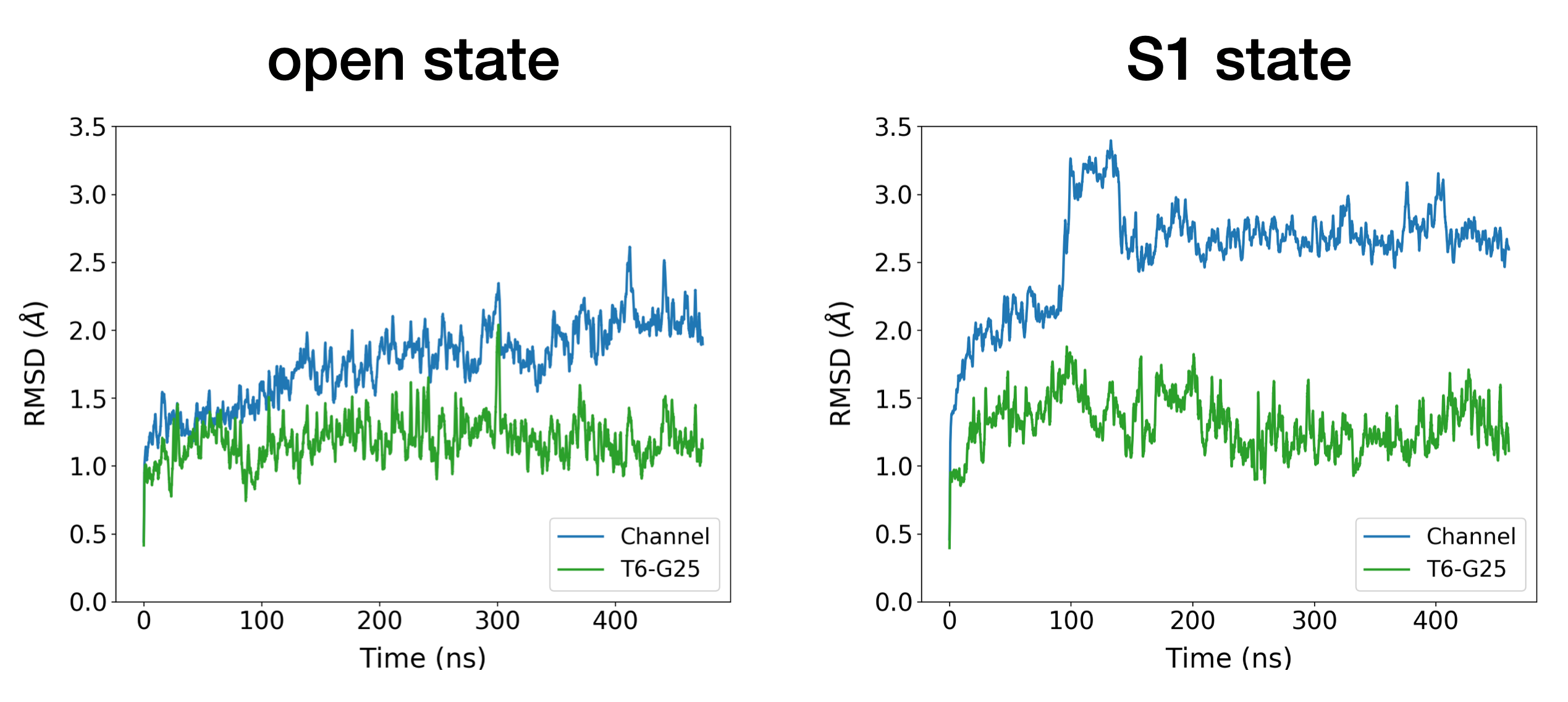

Supplement: S8 Fig — The backbone RMSD of the whole channel as well as the RMSD of the T6-G25 segment are shown. (TIF) [file pcbi.1008750.s008.tif]

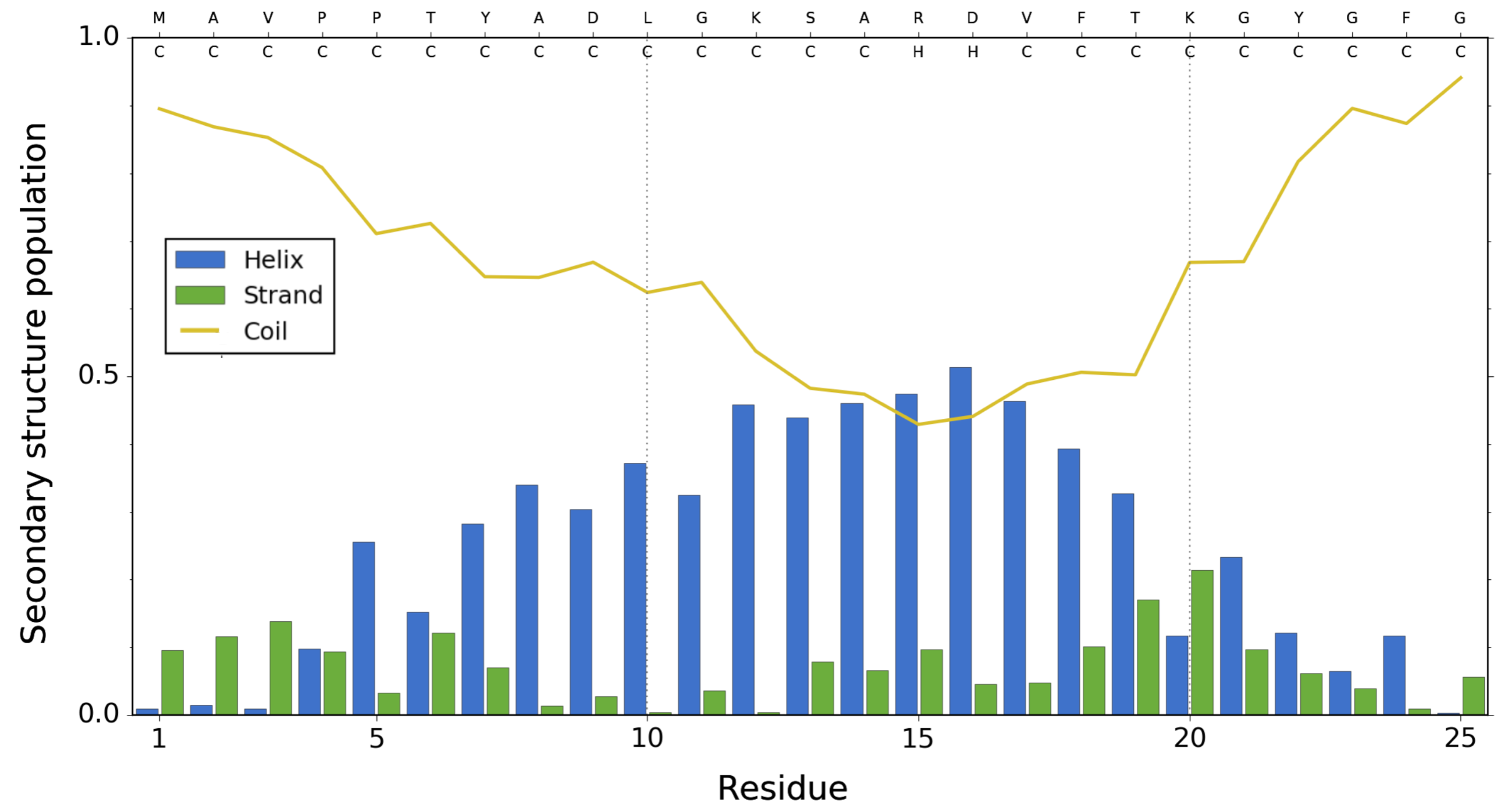

Supplement: S9 Fig — The graph displays random-coil and secondary structure propensity for each residue. (TIF) [file pcbi.1008750.s009.tif]

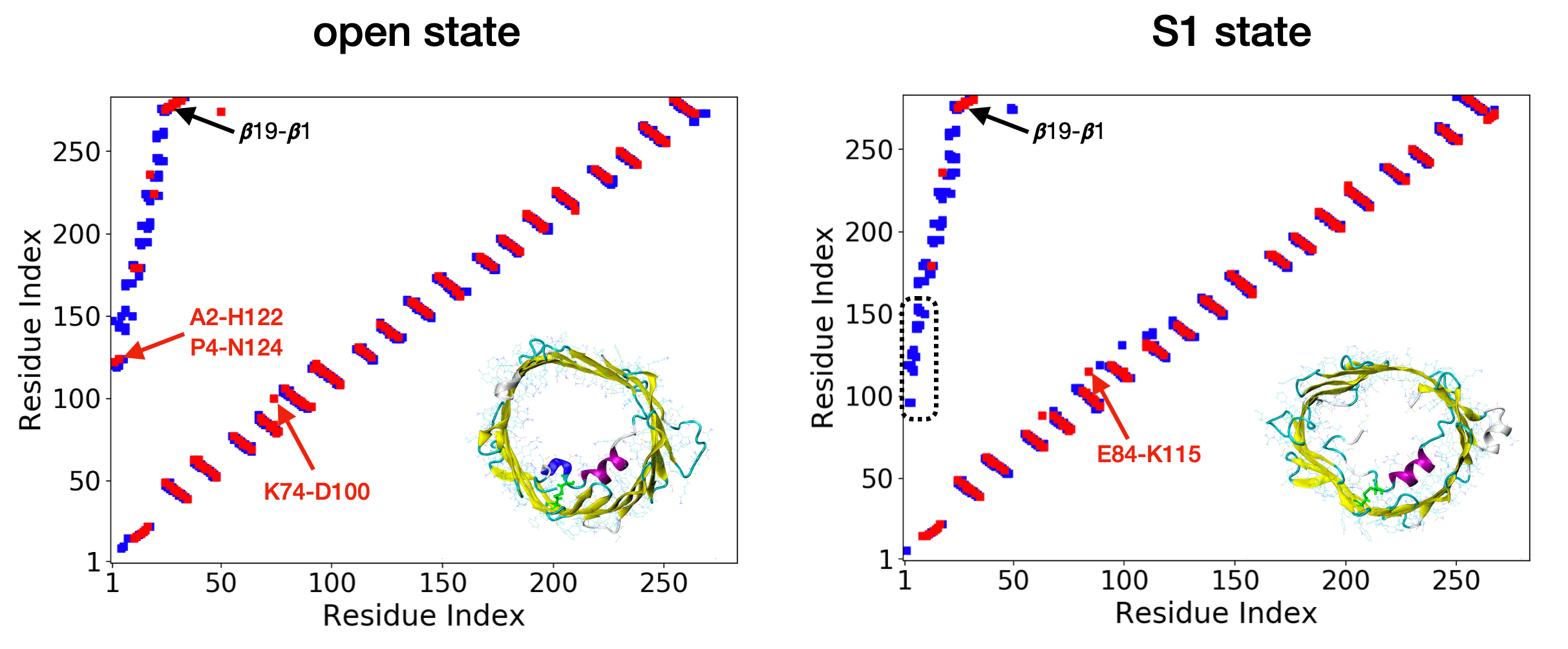

Supplement: S10 Fig — Each graph was built out of 25 frames equally spaced in time in each 450-ns-long trajectory. Only contacts occurring in 50% of cases or more are depicted. Red squares correspond to hydrogen bonds while blue squares stand for hydrophobic contacts. Note that contacts are only displayed in the upper left corner of each graph to avoid redundancy. In S1 state, the dashed rectangle includes hydrophobic contacts that stabilize the N-terminus against the barrel wall. Other interactions such as the E84-K115 salt bridge are shown. (TIF) [file pcbi.1008750.s010.tif]

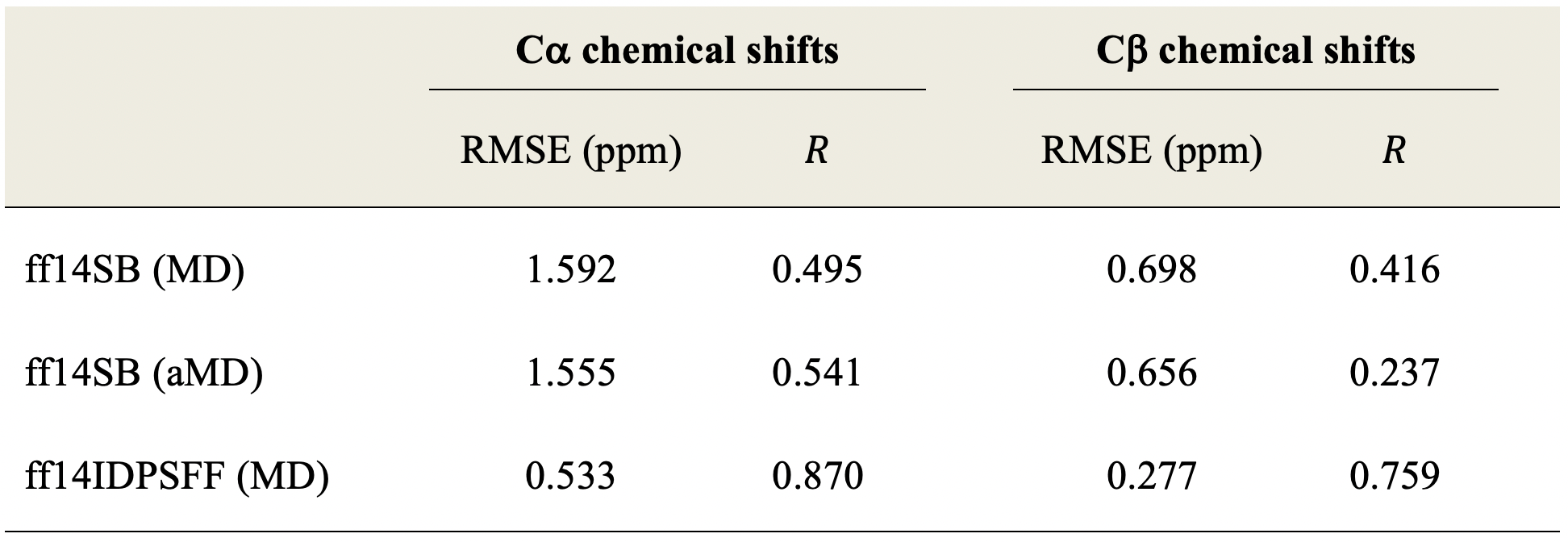

Supplement: S1 Table — Simulations include MD and aMD ones using either the ff14SB or the ff14IDPSFF force field. (TIF) [file pcbi.1008750.s011.tif]

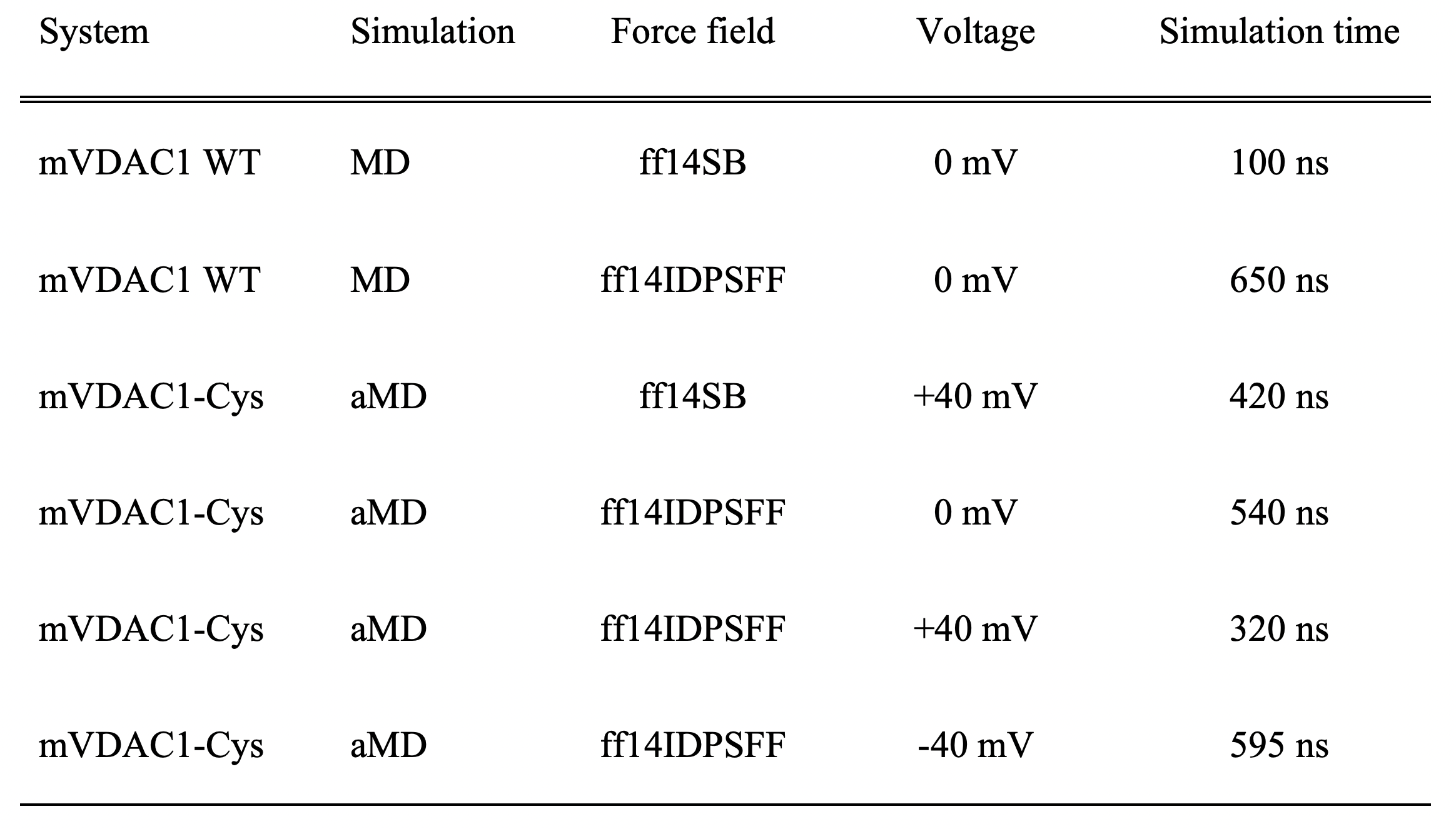

Supplement: S2 Table — (TIF) [file pcbi.1008750.s012.tif]

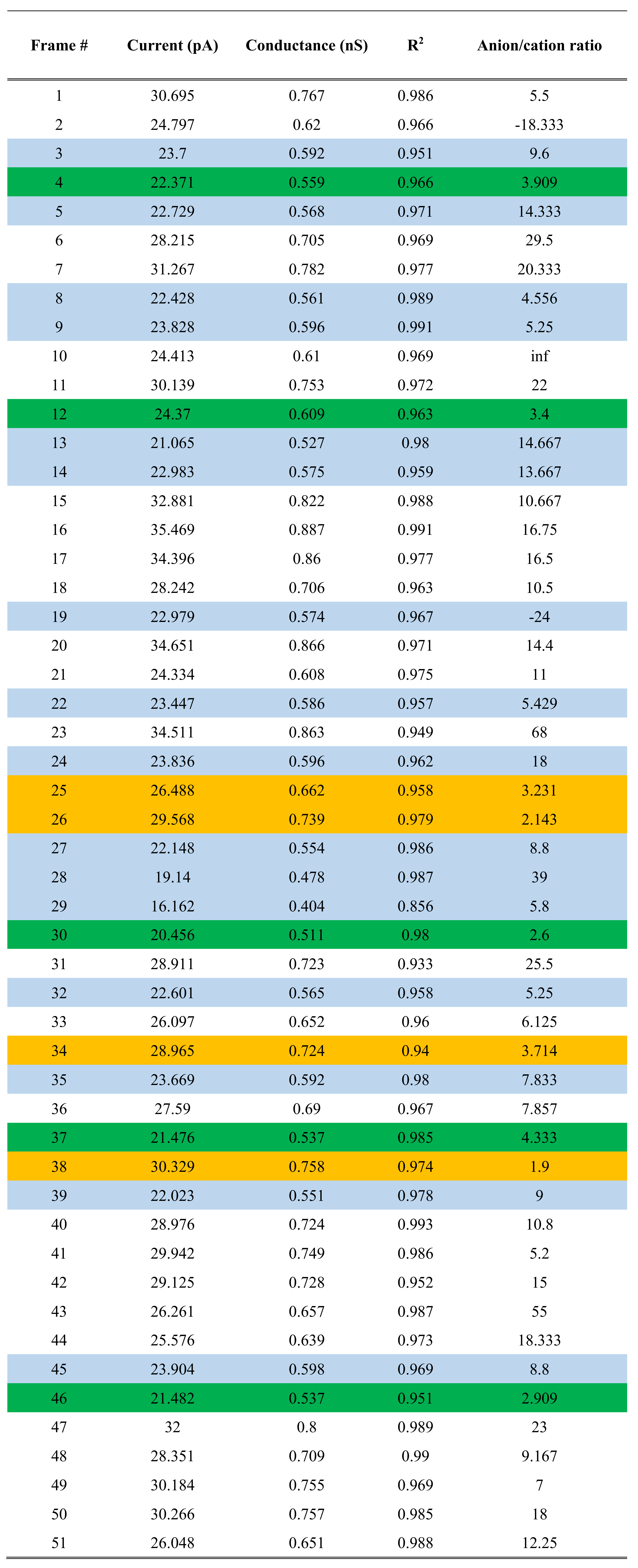

Supplement: S3 Table — The values were predicted by running 200-ns-long standard MD for each frame and by recording ion permeation events. The R2 coefficient obtained from linear regression of the crossing-events-vs-time curve is also displayed. The current was computed by multiplying the predicted slope of the curve by a scaling factor (see production MD (channel) in material and methods). Rows highlighted in green correspond to frames displaying both reduced conductance (around 0.6 nS or less) and a low anion/cation ratio (around 4 or less). Frames 30 and 46 correspond to the same structural S1 state while frames 4, 12 and 37 belong to S2 state (S7 Fig). Rows highlighted in orange stand for frames showing only low anion/cation ratio while rows in grey-blue are related to subconducting states with high anion/cation ratio (greater than 4.5). (TIF) [file pcbi.1008750.s013.tif]
