# Supplementary material for: The intrinsically disordered N-terminus of the voltage-dependent anion channel
Source: PLoS Comput Biol. 2021 Feb 12;17(2):e1008750. doi: 10.1371/journal.pcbi.1008750 (PMC7906469; doi:10.1371/journal.pcbi.1008750)
Supplement: Attachment — Submitted filename: responses_to_reviewers.docx [file pcbi.1008750.s021.docx]

**Responses to reviewers - point by point**

**Response to reviewer #2**

**Point 1:** However, there are some concerns regarding interpretation of L10C-A170C mVDAC simulations related to channel gating. It does not seem that the authors demonstrated consistent gating transition toward the closed state of the channel, which may happen on millisecond or even longer time scales (depending on the applied voltage and other factors). The authors observed partial unfolding of an untethered N terminal portion of the peptide under an applied voltage, which seems to lead to lower conductance and reduced selectivity based on GCMC/BD simulations they performed using all-atom aMD structures. It seems that these transitions may rather correspond to transient subconductance levels also mentioned in a previous study they cited (Briones et al Biophys J. 2016 Sep 20; 111(6): 1223–1234).

**Response:** We thank the referee for her/his comments. We are aware that gating transition occurs on the ms timescale (maybe longer). Although our aMD simulations were performed on hundreds of ns, effective timescales in aMD are expected to be much longer than in regular MD. The ability of aMD to account for transitions on the ms timescale from hundred-ns-long runs has been shown, e.g., on the 58-residue pancreatic trypsin inhibitor as mentioned in the first version of our paper (please see: *Pierce LC et al. Routine access to millisecond time scale events with accelerated molecular dynamics. JCTC 2012;8(9):2997-3002*). Remarkably, the pancreatic trypsin inhibitor is twice bigger than VDAC’s N-terminus so we believe that aMD could well capture subconducting transitions involving the latter. In addition, we found that standard MD of mVDAC1-Cys with the ff14IDPSFF force field was unable to escape from the basin associated with the 3EMN crystal structure which corresponds to our starting structure in our simulations. This confirms that the N-terminal segment stays in its folded state and that transition to a subconducting state **cannot** be observed over hundreds of ns using regular MD even when considering the disordered properties of the N-terminus. At the same time, we understand that the reviewer is questioning the metastability of our subconducting conformers generated at +40mV (*please note that the subconducting conformers generated at -40mV do correspond to transient levels as detailed in our discussion section*). That is why, we have included to the new version of the manuscript, standard MD simulations of our subconducting conformations generated from aMD. By recording the ion permeation events as a function of time, we were able to investigate the stability of each conformer in terms of conductance and anion/cation ratio. Among the 51 subconducting conformers^[1]^ identified in our +40mV aMD trajectory (between 240 ns and 290 ns), a few frames showed a stable subconducting behavior (~50-60% of the conductance in the open state) along with a low cation/anion ratio (~2-3) which prevail all along our hundred-ns-long MD runs (200 ns for each conformer, then 450 ns for the S1 state highlighted in the new version). For both open and subconducting trajectories, the estimated conductance and the anion/cation ratio were consistent with the results obtained from our previous GCMC/BD runs. The stability of our S1 state was also confirmed from the RMSD of both the N-terminus and the whole channel which was found to plateau early on in our new MD simulations (S8 Fig). Thus, the subconducting basin reported in our previous aMD simulation at +40mV does not correspond to transient levels as suggested by the reviewer but to metastable states that remain stable over *at least* hundreds of ns.

**Update:** we added a subsection (2.4) called “closed state(s)” where we introduced our new MD simulations and further structural analysis of the S1 state regarding the origin of the low conductance and the reduced anionic selectivity.

**Point 2:** Moreover, the authors did not seem to see (and/or analyzed) consistent changes in the rest of the voltage sensor, crucial for voltage-dependent transitions, as suggested in the experimental study they used as a basis of their simulations (Teijido et al J Biol Chem. 2012 Mar 30; 287(14): 11437–11445.): "During voltage-induced gating, the N-terminal region either stays inside the pore or moves in a concerted fashion with the rest of the voltage sensor rather than exerting large independent structural rearrangements to achieve voltage-dependent gating. It should be noted that the data presented here still permit the translocation of the N terminus in and out of the lumen upon gating, as suggested previously (16, 20, 32, 46), as long as this movement is coordinated with the voltage sensor."

**Response:** the paragraph cited by the reviewer was extracted from the discussion section in Teijido et al.’s paper. Notably, Teijido and coworkers have performed single-channel and multi-channel electrophysical measurements to show that the mVDAC1-Cys mutant had a gating profile similar to mVDAC1 WT. From this result, it was deduced that (i) VDAC’s closure could still arise while the N-terminus is tethered within the pore and (ii) that large conformational changes, i.e., those involving N-terminus’ translocation out of the lumen are not required to induce closure of the channel contrary to what has been previously suggested. Our results are in line with the discussion by Teijido and coworkers as the N-terminus always stayed inside the pore during our subconducting transition: “*During voltage-induced gating, the N-terminal region either stays inside the pore or moves in a concerted fashion with the rest of the voltage sensor rather than exerting large independent structural rearrangements to achieve voltage-dependent gating.*”. Obviously, the first scenario is what we found: “*the N-terminal region stays inside the pore*”. The second aspect to be discussed is the nature of the voltage sensor which, to our knowledge, has never been clearly elucidated. A few studies have suggested that the KRK motif located in the long alpha-helix of the N-terminus could play this role due to its (local) net positive charge (*Briones et al Biophys J. 2016 Sep 20; 111(6): 1223–1234*). However, no clear evidence has been reported so far.

**Update:** in the new section (2.4), we investigated the structural origin of the low conductance in our S1 state. Analysis of the density profile of Cl^-^ crossing events showed that the middle of the channel is partly blocked by the newly unfolded portion of the N-terminus (see new Fig 7). At the same time, an increase in K^+^ crossing events was also reported. The latter was found to be mainly located in the vicinity of residues E59, E73 and E84 which also suggests the involvement of these residues in the reduced selectivity.

**Point 3:** The structural changes the authors observed occurred on the time scale of only few to few hundred ns, many orders of magnitude shorter than physiological time scales for gating transitions. Although, effective time scales may be somewhat longer in aMD runs.

**Response:** as discussed in response to point 1, effective time scales in aMD are expected to be orders of magnitude longer (not “somewhat longer”) than those in regular MD.

**Point 4:** Since only singular, albeit pretty long aMD simulations were performed, there is also a possibility that presented trends may represent just random events rather than voltage-dependent changes. And this may be suggested by quite different outcomes of +40 and -40 mV simulations. Also, typically in MD simulations an applied voltage needs to be increased compared to physiological values to sample slow gating transitions during MD simulation time frame. So, using small physiological values is somewhat encouraging but again leaves a possibility of random voltage-independent events.

**Response:** we agree that smaller voltage values make it more difficult to dissociate between random events and voltage-induced ones. At the same time, we think that voltage values as high as 400 mV or 1 V (*Briones et al Biophys J. 2016 Sep 20; 111(6): 1223–1234, Noskov et al. Biochemistry, 52(51), 9246-9256*) may lead to extreme or unphysical behavior that would be unrepresentative of the real gating mechanism of the channel. In this regard, we believe that the use of biased techniques such as aMD – *which, again, enables to speed up of orders of magnitude the exploration of the conformational space* – at realistic voltage values, constitutes a more reliable approach. That being said, we understand the concern of the referee regarding random events vs. voltage-induced ones. Therefore, we have run 5 additional aMD trajectories (200 ns) at each voltage and measured the RMSD of the T6-D9 segment with respect to our S1 state as we showed that this segment was responsible for partially blocking the chloride flux. At +40 mV, 1 trajectory out of 5 was reported to reach conformers similar to S1 (RMSD < 3.0Å) while no such trajectories were found at 0 mV or -40 mV (RMSD > 4.0Å) suggesting that S1 might be typical of a positive voltage (see Fig 1 below). At the same time, we were not able to reproduce the S2 state in any trajectory. Although more thorough investigation is required to confirm the real impact of the electric field, stability of our S1 and S2 states over hundreds of nanoseconds already suggest their importance from a thermodynamic standpoint and that these conformers do not correspond to random transient structures.

**Fig 1**. RMSD of the T6-D9 segment with respect to S1 subconducting state highlighted in the new version. Calculations were carried for five 200ns-long trajectory replicas at 0mV, 40mV and -40mV.

**Update:** our aMD trajectory replicas are discussed at the end of the discussion section. Although our results are somewhat consistent with the S1 state being specifically induced by a positive voltage, we cannot rule out voltage-independent events. Another possible scenario being that high enough voltage – either positive or negative – would be especially important to make the N-terminus unfold (at least partially) and that transitions to closed states would arise in a more random way given the higher flexibility of the unfolded N-terminus. In that case, structurally-similar subconducting states may emerge regardless of the positivity of the voltage. Validation of one model over another is beyond the scope of the present paper whose primary objective is to point out that metastable subconducting states of mVDAC1-Cys can be generated at realistic voltage values by considering the intrinsically disordered properties of the N-terminus. Note that an additional phrase was added at the end of the introduction “*Although our subconducting states were generated at +40mV, the exact role of the membrane potential in channel closure, as compared with random voltage-independent events, is still to be determined*.”

**Point 7:** Also, related to this - how TM voltage was applied in simulations is not discussed.

**Response:** as detailed in section 4.8, the TM voltage in both aMD and MD simulations was introduced by applying a static electric field along the z direction.

**Point 8:** It should be also noted that even through CHARMM22+CMAP protein force field used in some of previous VDAC MD studies does not account explicitly for protein IDRs, this is done in the newest force field version - CHARMM36m (Huang et al Nat Methods 2017 Jan;14(1):71-73).

**Response/update:** we thank the reviewer for her/his comment. We added a few more lines in the discussion mentioning the CHARMM36m force field.

**Other suggestions and corrections:**

There are also quite a few specific suggestions and corrections:

p 7 It may help to make experimental data as thick lines in Fig 2

**Response:** done in the new version.

p 8 why aMD was not used for ff14IDPSFF simulations?

**Response:** aMD with ff14IDPSFF was not used on the N-terminal peptide as regular MD was found to be sufficient to reproduce experimental chemical shifts suggesting that most of the conformational landscape of the N-terminus has been visited. On the opposite, we were not sure that it was the case for ff14SB simulations as we observed discrepancies with experimental values. aMD was simply used to confirm that the discrepancies were not due to a sampling issue but were related to the force field itself.

p 9 "from S1 Table" -> "from Table S1"

**Response:** the “S1 Table” notation is specific of the journal. That is why we used it here. We agree that the “Table S1” notation is clearer.

p 10 l 208 "within the time frame of our standard MD (650 ns)" - However, 150 ns long MD simulation was mentioned earlier

**Response:** standard MD was run during 100 ns using the ff14SB force field and during 650 ns using ff14IDPSFF (see Table S2). Comparison between the two force fields was performed by using the first 100 ns of each trajectory (e.g., to plot Fig 3). Our ff14IDPSFF trajectory was simply extended to 650 ns to confirm the stability of the N-terminus helix-break-helix conformation in the open state, which was already well-known in the case of ff14SB. The reviewer referred to a 150-ns long MD simulation which was not mentioned in our manuscript.

p 13 Fig 4. It is not clear whether structures shown represent a snapshot at a particular simulation time (e.g. 10 ns) or some representative structures from the whole simulation. Also, it will be helpful if panels are labeled A and B or A, B, C and D. In the left panel, it will be helpful to show structures from different simulations by different colors or representations.

**Response:** old Fig 4 has been removed as the stability of the previous structures was not made clear (e.g., the conformer shown at -40mV in the old Fig 4 was only transient). However, we took into account the advices of the reviewer when making the new Fig 6. The structures displayed in Fig 6 correspond to representative structures obtained from 450ns-long MD runs. Labels A, B, C, D, E were used.

p 14 l 279 Why 0 mV MD simulations were not used for GCMC/BD runs at -40 mV, but used for runs at 40 mV.

**Response:** by running GCMC/BD simulations, our goal was to rapidly identify subconducting conformations generated in our aMD trajectory. As Ohm’s law was shown to apply in the case of VDAC (see *Choudhary et al. (2014). Nature struct. mol. Biol., 21(7), 626-632* and old Fig 6), the +40mV voltage was arbitrarily chosen to estimate the conductance of the conformers generated in the 0mV aMD trajectory.

p 14 l 283 "given at" -> "of"

**Response:** fixed in the new version

p 15 l 304 "Time curves" -> "Time series curves"

**Response:** fixed in the new version

p 16. l 311 "leading to slight compression of the latter in the region of interaction" Where such compression is demonstrated?

**Response:** The word “compression” was replaced by “apparent distortion” which, we think, will be less misleading to the reader. The locality of the distortion (yet still visible, see, e.g., Cluster #1 in S5 Fig) make it hard to quantify using, e.g., barrel’s ellipticity.

p 16 l 322 "Intensity" - current. The same in y axis labels in Fig 6.

**Response:** old Fig 6 was removed as GCMC/BD results are not necessarily representative of the stability of the structures. We believe that a figure like the new Fig 5 which shows the electric current as a function of time, is more appropriate to show the difference between the open and our subconducting state.

p 16 Fig. 6 Right panel legend should be moved to the center not to obstruct one of the curves. Labels A and B would be helpful.

**Response:** old Fig 6 was removed.

p 17 l 337 "unstructured properties" -> "unstructured conformation"

**Response:** in our opinion, talking about an unstructured conformation can be misleading as multiple unstructured conformers are expected in that case. In addition, no conformations were experimentally characterized by De Pinto and coworkers, only disordered properties were.

p 17 l 339 "G, A, S, P, R, Q, E and K" This list from ref. 30 includes amino acids enriched both fibrous and non-fibrous low-complexity proteins, and it's not clear whether the former are relevant for this work.

**Response:** although authors in Ref. 30 investigated both fibrous and non-fibrous proteins, the list still applies in the case of non-fibrous proteins only (see, e.g., graph A in Fig 3 of the mentioned reference which was made out of a set containing globular proteins only).

p 17 l 345 "N-terminus were disordered (not shown)." This along with at least one other secondary-structure prediction may need to be shown in Sup Info, as this is crucial for the study

**Response:** when using the ESpritz webserver, each amino acid is assigned a “O” or “D” letter depending on whether it is predicted to be involved in an ordered or disordered segment, respectively. However, no quantitative score is given as an output. Therefore, showing that all the amino acids are assigned a D letter does not provide much information. As suggested by the referee, we tested the N-terminal sequence using another algorithm, namely, the s2D method from the Vendruscolo lab which can predict both secondary structures and intrinsic disorder. s2D predicted that almost all the amino acids of the N-terminus were disordered although alpha-helix propensity was also observed especially for residues in the long alpha-helix (K12 to T19). The output graph generated from the s2D webserver is provided as S9 Fig.

p 18 l 374 "made of" -> "creating"

**Response:** we changed it by “characterized by” instead.

p 18 ll 375 "As shown by Teijido et al. (14), such an experiment enabled to

**Response:** it seems that this comment was not finished.

p 19 l 383 "stable interacting" -> "stably interacting"

**Response:** fixed in the new version.

p 19 l 391 "few" -> "a few" ?

**Response:** few is what we meant. Almost no subconducting structure was observed at 0 mV.

p 21 l 449 "MD production" -> "production MD"

**Response:** done.

p 25 l 533 It is not clear where PB/PNP was used

**Response:** A few lines regarding the PB/PNP method were added in section 4.9.

pp 25-26 ll 538-539 "500 clusters resulting in 500 frames were generated" - did each cluster contain just one structure or center of each cluster was selected?

**Response:** the center of each cluster was selected. The phrase was reformulated to make it clearer.

p 28 l 572 S4 fig. caption "wild-type mVDAC1" -> "wild-type mVDAC1 N-terminus"

**Response**: fixed in the new version.

p 28 ll 578-588 Figs S5 and S6 captions. Not only a disulfide bond, but also bonded Cys residues are shown in green, and H bonding residues seem to be shown in pink as well. Also, there is no similar figure for +40 mV simulation.

**Response**: S5 and S6 Figures were replaced by S5 Fig which includes representative structures of each simulation.

p 29 l 593 "Time curves" -> "Time series curves"

**Response**: fixed in the new version.

p 29 l 599 Table S1. Is it R or R^2, as more commonly reported? same for Table itself.

**Response**: R^2 is more commonly used for regression models, we simply computed R to estimate the linear correlation between simulated NMR shifts and experimental ones.

p 29 l 602 "Characteristics" -> "Parameters"?

**Response**: we have removed “characteristics” and simply wrote “Molecular simulations of mVDAC1 (WT and double Cys mutant) performed in the present study” as the legend of S2 Table.

Fig S2 can be a main text Figure

**Response:** in our opinion, the addition of S2 Fig in the main text will overload the manuscript in terms of figures. We made the choice of leaving the Figure in the SI.

Figs. S5 and S6 - % of two lower conformations are not shown Also, there is no similar figure for +40 mV simulation.

**Response**: S5 and S6 Figures were replaced by S5 Fig which includes clusters from the three trajectories.

Fig S7. Contact map is really confusing. I would just focus on the left corners and exclude adjacent residue interactions, which are the same. And in my opinion time series plots of most important interactions with labelling low-conductance states would be much more informative. And 0 mV simulation can be added in that case as well.

**Response**: Contact maps were redone in S10 Fig from 25 frames selected from our 450ns-long MD trajectories in the open and S1 states. Only contacts appearing in 50% of cases or more were shown. Important interactions or interaction areas are also more clearly pointed out.

Throughout the text it is confusing to see e.g. "S1 Figure" or "S2 Table" instead of "Table S1" or "Figure S2"

**Response**: again, the “S1 Figure” notation is characteristic of the journal.

**Response to reviewer #3**

We would like to thank the referee about her/his comments regarding the manuscript.

A few comment to improve the paper:

- From fig. 4 we see a deformation of the barrel, from spherical to elliptical. This is perhaps not due only to the double cysteine mutant, it was already observed in simulation of WT VDAC-1, as described in the paper Amodeo, G. F., et al. PLoS ONE 9, e103879 (2014). The author should comment on it.

-Not sure about their conclusion: “no apparent increase in the ellipticity of the barrel was reported during the formation of our subconducting states...” It seems to me that in fig. 4 the conformers 3 and 4 (from left to right) have a different ellipticity than 1 and 2. See also my previous comment. Perhaps the collapse is only minimal, however it might be induced by the presence of the disordered state of the N-terminal. They accelerated only some collective variables. I would expect that the rearrangement of the barrel toward a more elliptical stable state might occur on time scale not reached by the present simulations.

**Response:** indeed, the change in ellipticity was found to be minimal. The structure (in old Fig 4) mentioned by the reviewer corresponds to one of the conformers of the S2 state highlighted in the new version. As discussed in the new version (discussion section), calculations of mean ellipticity values revealed only small increases in the S2 or S1 states compared with the open state. While more thorough investigation is needed to explore whether the ellipticity is partly responsible for the low conductance, the observed increase in S2 is well below that of semicollapsed states reported in Ref. 11, i.e., Zachariae U et al. (2012), Structure 20(9):1540-1549. In addition, we would like to comment on the statement “They accelerated only some collective variables” which can give the (wrong) impression that we only accelerated specific atoms or residues of the system. Although it is true that aMD is based on a biased potential which is energy-dependent (i.e., the collective variables are the total and dihedral energies), all the atoms of the system are impacted by the bias. Therefore, aMD could, in theory, be used to accelerate the transition to stable highly-elliptical states if any. However, no such states have been reported in our study. Nevertheless, given the multiplicity of VDAC’s closed states, we leave open the possibility that other stable closed states exhibiting high ellipticity, could exist.

**Update:** the old paragraph regarding ellipticity was replaced by a new one in the discussion section which includes the elements discussed above.

It would be interesting to see the path of ions in CGMC/BD simulations, in two representative of both the closed and open states.

**Response/update:** we would like to thank the referee for her/his useful remark. Instead of representing the path of ions which may be difficult to identify as ions cross the channel at many different positions at fixed z, we checked the (x, y) coordinates of crossing events at the middle of the channel (Fig 7 in the new version of the manuscript). From this representation, the origin of the subconducting behavior as well as the decrease in anion selectivity became more apparent as discussed in the new version.

1. Those conformers correspond to the frames characterized by a conductance less than 0.6 nS as predicted from our original GCMC/BD simulations
