# Supplementary material for: The intrinsically disordered N-terminus of the voltage-dependent anion channel
Source: PLoS Comput Biol. 2021 Feb 12;17(2):e1008750. doi: 10.1371/journal.pcbi.1008750 (PMC7906469; doi:10.1371/journal.pcbi.1008750)
Supplement: Attachment — Submitted filename: responses_to_reviewers.docx [file pcbi.1008750.s022.docx]

**Responses to reviewers - point by point**

**Response to reviewer #2**

We would to thank the reviewer for her/his general comments regarding our manuscript, which are very much appreciated. We also would like to thank her/him for her/his thorough work in reviewing our paper, which has definitely contributed to improving its quality. As the reviewer can see in the new submitted version, we have revised our manuscript according to her/his last minor suggestions. We also made minor wording changes in a few sentences to make them more understandable to the reader.

**Minor points:**

p 4 l 85 "Chloride and potassium ions" - please specify colors
p 5 l 109 "cis side" - please specify more explicitly or omit to avoid confusion as it's discussed further in the text
p 7 "beta-carbons chemical shifts" -> "beta-carbon chemical shifts"
p 8 l 172 "ff14SB, were" -> "ff14SB were" (remove comma)
p 9 l 180 "overexpress" -> "overstabilize"
p 12 l 251 "hydrogen bonds at" -> "hydrogen bonds between"
p 12 l 255 "Bonded" -> "Disulfide bridge linked"
p 12 l 256 "IMS view" Maybe also specify top or bottom relative to structure shown in Fig. 1 and/or specify in Fig. 1, which one is IMS/cytoplasmic side and also maybe cis/trans sides.
p 13 l 260, 261"hydrogen bonds at" -> "hydrogen bonds between"
p 13 l 264 "hydrophobic patch" -> "hydrophobic interaction or contact"
p 19 l 371 "around twice smaller" -> "about twice as small as"
p 12 l 374 "Bonded" -> "Disulfide bridge linked"
p 19 l 419 "hydrophobic patch" -> "hydrophobic interaction or contact"
p 22 l 443 "experimental shifts" -> "experimental chemical shifts"
p 24 l 484 "K+" -> "K^+"
p 25 l 511 "can be prevail at" -> "can prevail at"
p 26 l 543 "MD production" -> "production MD"
p 30 l 616 "150nM KCl" -> "150 mM KCl" ? 150 nM is a very low, not physiological concentration and not possible to model via MD in most cases
p 30 l 628 "at 10nM" -> "at 10 mM"? please see comment above
p 31 l 648 "on each point" -> "at each point"
p 31 l 650 "5. 10^7" -> "5x10^7"
p 32 ll 661, 667 "of the N-terminal peptide." -> "of the VDAC1 N-terminal peptide."
p 32 ll 664, 670 "N-terminal conformation" -> "N-terminal peptide conformation"
p 32 l 678 "regular helix content" -> "regular alpha-helical content"
p 32 l 683 "Bonded" -> "Disulfide bridge linked"
p 32 l 685 Please specify viewpoint e.g. with respect to Fig. 1top/bottom and/or IMS/cytoplasmic
p 33 l 695 same as above
p 33 l 698 "of the s2D method" -> "of the sequence-based protein secondary structure prediction s2D method"
p 33 l 700 "open state and S1" -> "open and S1 states"
p 32 l 707 RMSE -> "Root-mean-square error (RMSE)"
p 34 l 708 "of the N-terminal peptide." -> "of the VDAC1 N-terminal
p 34 ll 725, 728 "Bonded" -> "Disulfide bridge linked"
p 35 ll 732, 736, 740, 742 "Bonded" -> "Disulfide bridge linked"
pp 34 - 35 Movie S1-S7 captions. Please specify viewpoints e.g. with respect to Fig. 1 top/bottom and/or IMS/cytoplasmic. You may also specify color scheme used for alpha-helix, 3-10 helix, beta-barrel etc. or refer to previous figures.

**Update:** All the points were fixed in the new version. Note that cytoplasm and IMS sides were specified in the new Fig 1 and captions related to Movie S1-S7 were improved according to suggestions.
